# Quality improvement program in radiation oncology: understanding patient hospitalizations, treatment breaks, and weight loss in patients receiving radiotherapy

**DOI:** 10.1186/s13014-018-1111-1

**Published:** 2018-08-29

**Authors:** Varun Chowdhry, Pamela Jangro, Saveli Goldberg, Nolan Gagne, Shelley Chabot, Gary Proulx, Cocav Rauwerdink

**Affiliations:** 1 Department of Radiation Medicine, Roswell Park Comprehensive Cancer Center, Buffalo, NY USA; 2 Department of Radiation Oncology, Exeter Hospital, Exeter, NH USA; 3 Division of Hematology/Oncology, Massachusetts General Hospital, Boston, MA USA

## Abstract

**Introduction:**

The reporting of adverse effects is an integral aspect of a hospital quality improvement (QI) program with the goal of improving care for current and future patients. We report the results of our experience tracking patient hospitalizations, treatment breaks, and weight loss in patients receiving radiotherapy as part of a departmental QI program.

**Methods:**

In 2014, the Center for Cancer Care at Exeter hospital developed a departmental quality initiative to track adverse outcomes in a population of patients receiving radiation therapy. Criteria for inclusion in this initiative included: treatment break ≥3 days, hospitalization either while on treatment of within 2 weeks of treatment, death within 2 weeks of treatment, or weight loss of ≥10%. Patients included on this registry were reviewed at regularly scheduled departmental QI meetings, where solutions for improvement were discussed.

**Results:**

Ninety-one patients were identified as having an event that meet the above-mentioned criteria. Forty-three patients were receiving concurrent chemotherapy (47.2%) Fifty-four (54.9%) patients had toxicity directly attributable to their treatment. Sixty-five patients (71.4%) were treated with curative intent. Nineteen patients (21.1%) died either during the course of radiotherapy, or within two weeks of completion of treatment. Advanced age was significantly associated with inferior overall and disease free survival in this analysis, HR 1.030 (1.006–1.054) *p* = 0.0125, and HR 1.034 (1.008–1.061) *p* = 0.010 respectively.

**Conclusion:**

We believe that this protocol to track events has been helpful in making practice changes in our department. Our results suggest that elderly patients who experience qualifying event are at increased risk of death, and providers should be cognizant of this finding. Future QI projects can seek to better understand how such changes have resulted in improvements in patient care.

## Introduction

The concept of a morbidity and mortality conference is well known to physicians number of different medical specialties, yet the exact definition of what constitutes such an incident is not always well defined [1]. Oncological care poses unique challenges as toxicity from a particular therapy may be somewhat predictable, yet patients’ experience of this toxicity can range on a spectrum. While the American College of Radiation (ACR) recommends that individual departments track morbidity and mortality data as part of continuous quality improvement (CQI) [2], there is no specific standard with regard to how this information should be reported. There has been recent interest in utilizing this information and incorporating solutions for improvement [3]. Our Quality Improvement project focused on obtaining information from several of these parameters pertinent to our specific practice. While many institutions utilize a case-based system as part of quality improvement (QI), we report our experience of systematic tracking of defined metrics.

## Materials and methods

In 2014, the Center for Cancer Care at Exeter Hospital began a QI initiative to better track and identify patients who may have experienced an adverse event either during or immediately after a course of radiotherapy. The parameters selected were based on ACR guidelines [2] of tracking morbidity and mortality, and departmental clinical experience as to important events that merited further discussion. Criteria for inclusion in this initiative included: treatment break ≥3 days, hospitalization either while on treatment of within 2 weeks of treatment, any death on treatment or within 2 weeks of treatment. In 2017, the metric was expanded to include patients who experienced a weight loss of ≥10%. This metric was specifically added to track patients who were at risk of hospitalization. Our department felt that such patients could potentially benefit from nutritional consultation, IV hydration, and consideration of feeding tube placement, as clinically appropriate. In addition, patients with any other known significant acute or late complications were included, as well as any patient who experienced a radiation misadministration. Only patients included on this departmental registry were included in this analysis. Data for events was obtained through information obtained in weekly treatment visits, as well as review of hospital reports for patients who were admitted to the hospital. Patients were reviewed at regularly scheduled departmental QI meetings with members of the care team present, including physicians, physicists, dosimetrists, nursing, social worker and radiation therapists, where solutions for current and future improvement were discussed.

## Results

**Fig. 1:**
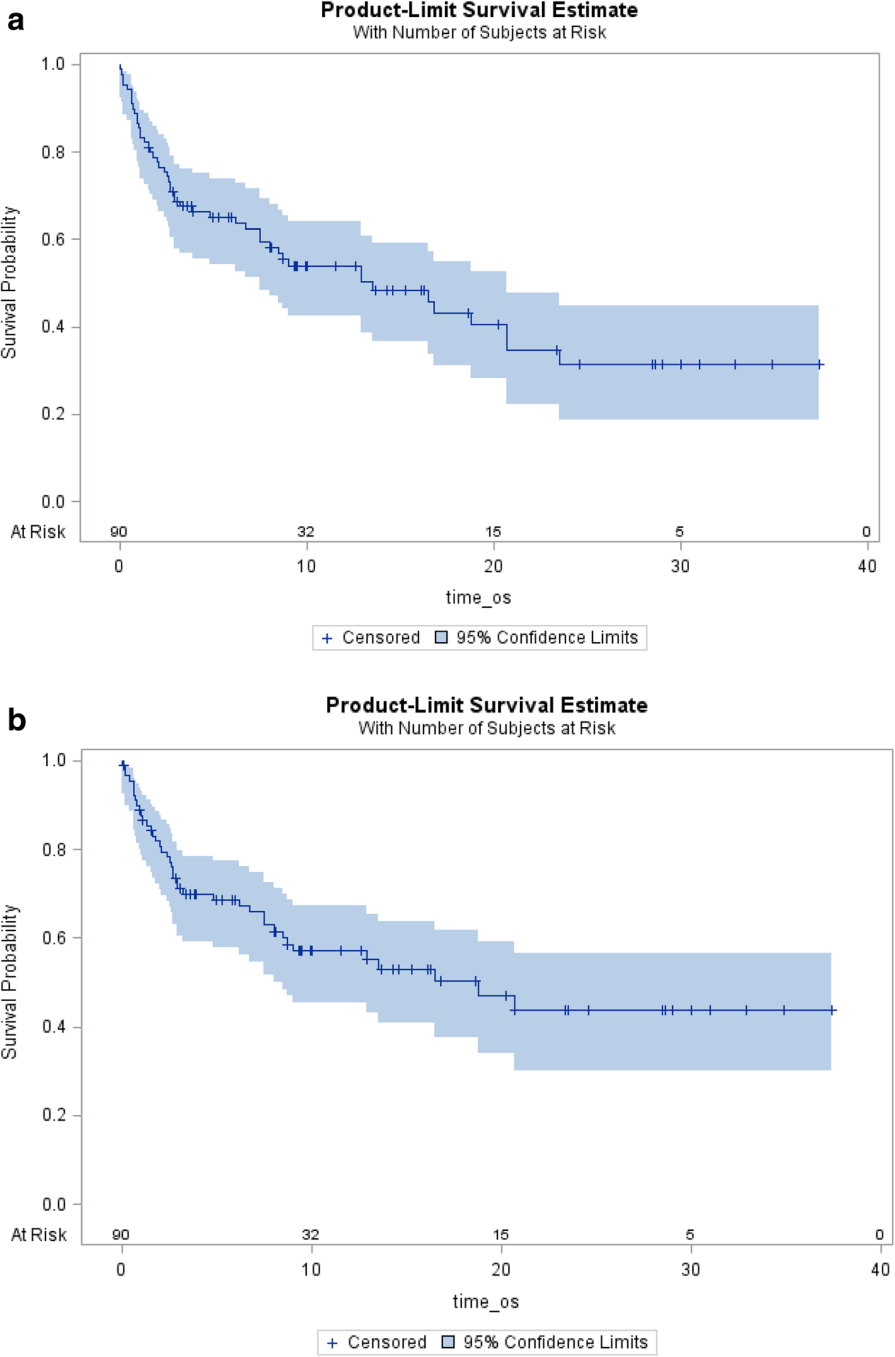
**a** Overall Survival: Median Overall Survival is 13.5 months (95%CI: 7.5–20.7). At 1 year 54.0% (95%CI:42.6–64.0). **b** Median Disease free survival was 18.8 months. Disease free survival was one year was 57.1% (95%CI:45.3–67.2)

General information regarding our facility is listed on Table 1. The Center for Cancer Care at Exeter Hospital, is a community based cancer center that administers an average of 8000 treatments each year. Demographic information for patients included in this registry are reported in Table 2. A total of 91 patients were identified as having an event that met the above mentioned criteria. Median overall and disease free survival are reported in Fig. 1a and b respectively. Median survival for patients on this registry was 13.5 months (95%CI: 7.5–20.7), overall survival at one year was 54.0% (95%CI:42.6–64.0). Forty-three patients were receiving concurrent chemotherapy (47.2%) Fifty-four (54.9%) patients had toxicity directly attributable to their treatment. Sixty-five patients (71.4%) were treated with curative intent. Nineteen patients (21.1%) died either during the course of radiation, or within two weeks of completion of treatment. Breakdown of patients included by disease site is included in Table 3. The most common disease site of patients included on this registry was patients treated for head and neck cancer, 26 patients, 28.6% of patients. Breakdown by histological diagnosis is reported in Table 4. In order to be included on this registry, patients had to meet one of the above mentioned criteria. The most common reason for being included on this registry was skin toxicity (17 patients, 18.7%), resulting in a treatment break.

**Table 1:** Demographic information

New Patient Consultations	468
Radiation Treatment Visits	8073
Radiation Misadministration	0
Average Daily Fall Risk	3.6
Total Number of Patients	91
Male	51 (56.0%)
Female	40 (44.0%)
Median Age (years)	67 (range, 26–92)
Median Planned Radiation Dose (cGy)	5940 (range, 2250–7440)
Median Actual Radiation Dose (cGy)	5040 (range, 300–7440)
Treatment Intent	
Curative	65 (71.4%)
Palliative	26 (28.6%)
Death within 2 weeks of radiation completion	19 (21.1%)
Number of Patients with Treatment Break ≥3 days	58 (63.7%)
Median Duration of Treatment Break (days)	5

**Table 2:** Breakdown by radiation treatment site

Head and Neck	26	28.6%
Lung/Thorax	13	14.3%
Breast/Chest Wall	10	11.0%
Pelvis/Anal/Rectum	10	11.0%
CNS	9	9.9%
Esophagus	7	7.7%
Spine/Bone Met	7	7.7%
Skin/Extremity	4	4.4%
Bladder	2	2.2%
Abdomen/Pancreas	2	2.2%
Gynecological	1	1.1%
Prostate	0	0.0%

**Table 3:** Reason for inclusion

Skin	17	18.7%
Hematologic	14	15.4%
Disease Progression/Treatment Ineffective	12	13.2%
Patient Death	8	8.8%
Admitted to Hospital For Non-Oncological Reason	7	7.7%
Excessive Pain	6	6.6%
Weight loss	6	6.6%
Pulmonary (pneumonia/COPD)	4	4.4%
Gastrointestinal (nausea, vomiting, diarrhea)	4	4.4%
Head and Neck (Mucositis)	4	4.4%
Neurological (CVA)	3	3.3%
Patient Non-compliance/social issues	3	3.3%
Fall	2	2.2%
Other side effects	1	1.1%

**Table 4:** Breakdown by histology

Squamous cell carcinoma	34	37.4%
Adenocarcinoma	16	17.6%
Breast cancer (ductal or lobular)	11	12.1%
Carcinoma, NOS	7	7.7%
Small cell carcinoma	4	4.4%
Lymphoma	4	4.4%
Sarcoma	3	3.3%
Basal cell carcinoma	3	3.3%
Melanoma	2	2.2%
Transitional cell carcinoma	2	2.2%
Urothelial carcinoma	1	1.1%
Glioblastoma	1	1.1%
Multiple myleoma	1	1.1%
Neuroendocrine carcinoma	1	1.1%
Thymoma	1	1.1%

Advanced age was significantly associated with inferior overall and disease free survival in this analysis, HR 1.030 (1.006–1.054) *p* = 0.0125, and HR 1.034 (1.008–1.061) *p* = 0.010 respectively.

## Discussion

In this quality initiative, we recorded events we believe to be clinically significant and warrant detailed review in a multidisciplinary and intradepartmental setting. Since patients who experience prolonged treatment breaks have inferior clinical outcomes compared to patients who experience uninterrupted courses of treatment [4, 5], we believe it is important to critically evaluate each occurrence. Reviewing each event as a group, and creating greater awareness of potential adverse events due to radiotherapy and chemotherapy have directly and indirectly resulted in practice changes. Our group has become aggressive about the initiation of supportive care prior to the start of treatment. For example, in our multidisciplinary head and neck cancer conference, we arrange for patients to meet with a nutritionist and speech therapist at the time of initial consultation. Additionally, we now encourage placement of a feeding tube for patients at high risk of mucositis to prevent dehydration and hospitalization. Aggressive multidisciplinary care has been shown to improve clinical outcomes in patients being treated with several different malignancies [6, 7], and we believe such interventions have reduced the frequency and duration of toxicity in our population.

This initiative has also led to practice changes in our management of skin toxicity. Patients felt to be at high risk for experiencing an adverse skin reaction are often referred to wound clinic immediately at the time of consultation. For example, patients with significant edema, or large tumor creating a non-healing wound are referred to would care clinic prior to the start of treatment. We have also noticed that changes to our treatment planning paradigm in patients with breast cancer has resulted in fewer treatment breaks.

In 2017, we began to include patients who experienced weight loss ≥10% has been helpful to identify patients at higher risk of clinical dehydration and hospitalization, and has been helpful to recognize patients who may need modifications in radiation treatment plan due to weight changes.

We believe that reviewing patient death during a course of radiation treatment has contributed to changes in clinical practice. As patients receiving palliative radiotherapy advanced cancer are at a high risk of short term morbidity and mortality, we have increased our utilization of single fraction radiation dose regimens, which have been shown to result in equivalent level of pain control [8], while reducing the number of visits to the doctor. Additionally, creating a cultural awareness to discuss such events in our department may have contributed to our increased our utilization of early palliative care, which has been shown to improve survival for patients with Stage IV non-small cell lung cancer in a randomized clinical trial [9].

Our finding that age is significantly associated with inferior overall and disease specific survival also warrants further discussion. While older patients in this registry may be more likely to have advanced malignancies and thus at an increased risk of death, finding suggests that special attention should be provided to this population of patients.

The fact that the advanced age was associated with an inferior rate of disease free and overall survival warrants further discussion. We can hypothesize potential explanations. An elderly patient is more likely to have other medical comorbidities which could make it more difficult to complete definitive therapy, potentially resulting in inferior disease free survival. In addition, patients with advanced age may have been more likely to be treated for palliative as opposed to definitive indications further confounding the data. Nevertheless, the information is certainly hypothesis generating, and this population certainly warrants special attention.

It is important to recognize potential shortcomings of our initiative, and potential for future study. Our data is self-collected, and we do not have a comprehensive way to collect every potential incident. While we do our best to track potential adverse events by reviewing hospital reports and following patients who completed treatment; we must acknowledge that some instances of toxicity that can be missed. For example, we could be limited in our ability to gather data in a patient who is hospitalized at an outside institution.

It is also important to recognize that our metric does not capture many forms of toxicity from radiotherapy; particularly late toxicity. Our metric is more sensitive for capturing patients with acute toxicity from treatment. For example, a patient with late cardiac event may be unknown to us, as it is often difficult to attribute late toxicity to a single cause. Future metrics may attempt to better track late toxicities from patients who are seen in a follow-up visit.

Another limitation of our metric is that while our quality project has resulted in practice changes, we do not have specific outcome data regarding how such changes have impacted patients. While we believe that such changes have improved patient care, we believe future quality studies could seek to capture metrics that can demonstrate improvements with regard to clinical outcome.

## Conclusion

Our system of tracking track adverse outcomes to include patients who experience a treatment break ≥3 days, hospitalization either while on treatment of within 2 weeks of treatment, any death during or within 2 weeks of treatment, or weight loss of ≥10% was a helpful QI initiative for a radiation oncology department that has resulted in changes in departmental practice. We believe that creating a forum to review adverse outcomes could be helpful to future patients. At the same time, our department should seek to capture additional metrics that may be helpful in quantifying how these changes have resulted in improvements in patient care. The finding that advanced age is associated with an inferior outcome suggests that elderly patients should be given close attention during treatment.

## Abbreviations

ACR: American College of Radiation
CQI: Continuous quality improvement
QI: Quality improvement
